# Contouring of emerging organs-at-risk (OARS) of the female pelvis and interobserver variability: A study by the Italian association of radiotherapy and clinical oncology (AIRO)

**DOI:** 10.1016/j.ctro.2023.100688

**Published:** 2023-10-06

**Authors:** A. Augurio, G. Macchia, L. Caravatta, M. Lucarelli, F. Di Gugliemo, A. Vinciguerra, B. Seccia, V. De Sanctis, R. Autorino, C. Delle Curti, S. Meregalli, E. Perrucci, D. Raspanti, A. Cerrotta

**Affiliations:** a Department of Radiation Oncology, SS. Annunziata Hospital, Via Dei Vestini, 66100 Chieti, Italy; b Radiation Oncology Unit, Gemelli Molise Hospital, Università Cattolica del Sacro Cuore, Largo Agostino Gemelli, 1, 86100 Campobasso, Italy; c Department od Radiotion Oncology, SS Annunziata Hospital, "G. D'Annunzio" University, Via dei Vestini, 66100 Chieti, Italy; d Department of Neuroscience, Imaging and Clinical Sciences, “G. D’Annunzio” University, Via Luigi Polacchi 11, 66100 Chieti, Italy; e Radiotherapy Oncology, Department of Medicine and Surgery and Translational Medicine, Sapienza University of Rome, S. Andrea Hospital, Via di Grottarossa 1035, 00189 Rome, Italy; f Oncological Radiotherapy Unit, Department of Diagnostic Imaging, Oncological Radiotherapy and Hematology, Fondazione Policlinico Universitario A. Gemelli IRCCS, Via Giuseppe Moscati, 31, 00168 Rome, Italy; g Radioterapia Oncologica, Fondazione IRCS, Istituto Nazionale dei Tumori di Milano, Via Giacomo Venezian, 1, 20133 Milano, Italy; h Radiotherapy Unit, Azienda Ospedaliera San Gerardo, Via G. B. Pergolesi, 33, 20900 Monza, Italy; i Radiation Oncology Section, Perugia General Hospital, Piazzale Giorgio Menghini, 3, 06129 Perugia, Italy; j Temasinergie S.p.A., Via Marcello Malpighi 120, Faenza, Italy

**Keywords:** Emerging female pelvic OARs, Contouring atlas, Interobserver variability, Radiotherapy, Gynecological cancer

## Abstract

**Purpose:**

To provide straightforward instructions for daily practice in delineating emerging organs-at-risk (OARs) of the female pelvis and to discuss the interobserver variability in a two-step multicenter study.

**Methods and materials:**

A contouring atlas with anatomical boundaries for each emerging OAR was realized by radiation oncologists and radiologists who are experts in pelvic imaging, as per their knowledge and clinical practice. These contours were identified as quality benchmarks for the analysis subsequently carried out. Radiation oncologists not involved in setting the custom-built contouring atlas and interested in the treatment of gynecological cancer were invited to participate in this 2-step trial. In the first step all participants were supplied with a selected clinical case of locally advanced cervical cancer and had to identify emerging OARs (Levator ani muscle; Puborectalis muscle; Internal anal sphincter; External anal sphincter; Bladder base and trigone; Bladder neck; Iliac Bone Marrow; Lower Pelvis Bone Marrow; Lumbosacral Bone Marrow) based on their own personal knowledge of pelvic anatomy and experience. The suggested OARs and the contouring process were then presented at a subsequent webinar meeting with a contouring laboratory. Finally, in the second step, after the webinar meeting, each participant who had joined the study but was not involved in setting the benchmark received the custom-built contouring atlas with anatomical boundaries and was requested to delineate again the OARs using the tool provided. The Dice Similarity Coefficient (DSC) and the Jaccard Similarity Coefficient (JSC) were used to evaluate the spatial overlap accuracy of the different volume delineations and compared with the benchmark; the Hausdorff distance (HD) and the mean distance to agreement (MDA) to explore the distance between contours. All the results were reported as sample mean and standard deviation (SD).

**Results:**

Fifteen radiation oncologists from different Institutions joined the study. The participants had a high agreement degree for pelvic bones sub-structures delineation according to DICE (IBM: 0.9 ± 0.02; LPBM: 0.91 ± 0.01). A moderate degree according to DICE was showed for ovaries (Right: 0.61 ± 0.16, Left: 0.72 ± 0.05), vagina (0.575 ± 0.13), bladder sub-structures (0.515 ± 0.08) and EAS (0.605 ± 0.05), whereas a low degree for the other sub-structures of the anal-rectal sphincter complex (LAM: 0.345 ± 0.07, PRM: 0.41 ± 0.10, and IAS: 0.4 ± 0.07).

**Conclusion:**

This study found a moderate to low level of agreement in the delineation of the female pelvis emerging OARs, with a high degree of variability among observers. The development of delineation tools should be encouraged to improve the routine contouring of these OARs and increase the quality and consistency of radiotherapy planning.

## Highlights

• Damage to the anatomical substructure caused by radiotherapy is currently recognized as the cause of pelvic toxicity.
• Newly emerging OARs of the female pelvis must be recognized and delineated.
• Interobserver variability in the contouring of emerging female pelvic OARs is high.
• A contouring atlas with anatomical boundaries for the emerging female pelvic OARs is provided.

## Introduction

Radiotherapy (RT) is currently considered a cornerstone in management of pelvic female tumors. Among gynecological malignances, cervical cancer represents the fourth most common cancer globally and is one of the leading causes of cancer-related morbidity and mortality in women [1]. Definitive external beam radiation therapy (EBRT) with concurrent platinum-based chemotherapy followed by brachytherapy (BT) is the standard of care for locally advanced cervical cancer (Locally Advanced Cervical Cancer (LACC): IB3–IVA FIGO 2018 definition).

Modern RT as image-guided radiation therapy (IGRT) with IMRT and/or BT have been shown to improve outcomes in LACC patients with a significant reduction of grade ≥ 2 acute and late toxicities [2], [3]. Since the ability of IMRT to deliver highly conformal radiation to target volumes and minimize dose to organs-at-risk (OARs), an accurate delineation of volumes of interest needs to be performed. A consensus guideline for pelvic normal tissue contouring on a CT image atlas has been endorsed by RTOG to allow uniformity in defining normal tissues [4]. Afterwards, the GEC-ESTRO working group prompted a standardization of volumes, prescribing and reporting doses for IMRT and IGBT [5], [6], [7], [8], and in association with the American Brachytherapy Society (ABS), recommendations to generate a composite Radiation Biologically Effective Dose (BED) have been provided [9]. Although Magnetic resonance Imaging (MRI) is the gold standard for IGBT, due to the low widespread in resource limited settings, the IBS-GEC-ESTRO ABS has recently proposed new consensus guidelines to facilitate accurate CT-based contouring following the anatomical boundaries [10]. The main OARs in routine CT based delineation are pelvic bones, rectum, bladder, and bowel, while the anal canal, vagina and urethra are recommended only when target volume and dose distribution are in proximity. Moreover, the damage induced by RT to anatomic sub-structure (i.e., anal sphincter complex, bladder base and neck, and vagina) is currently recognized in the pathogenesis of pelvic acute and late toxicities [11], [12], [13], [14], [15]. The identification of novel structures as emerging OARs may be recommended as a result of the possibility of avoiding dosage to OARs using modern RT techniques and the availability of MRI-based contouring. In this context, we proposed, together with the Italian Association of Radiotherapy and clinical Oncology (AIRO) Gynecology Study Group, a multi-institutional 2-steps study to assess conformity grade in delineation of emerging OARs of female pelvis, starting from a benchmark contouring atlas with anatomical boundaries realized by radiologists and radiation oncologists who are specialists in pelvic imaging. The aim of this paper is to provide straightforward instructions for daily practice in delineating emerging OARs of the female pelvis and to discuss the interobserver variability in a two-step multicenter study.

## Methods

### Study design

A custom-built contouring atlas with anatomical boundaries for each emerging OAR was realized by the Principal Investigator [AA] and a team of radiation oncologists [LC, AC, GM, AV] and radiologists [BS] dedicated to pelvic imaging, as per their knowledge and clinical practice. These contours were identified as quality benchmarks for the two-step analysis subsequently carried out. The two-step contouring study was performed between January 2022 and September 2022 at the Department of Radiation Oncology of the University “G. D'Annunzio” (Chieti) to investigate the inter-observer variability in the delineation of new-emerging OARs of the female pelvis. Radiation oncologists not involved in setting the custom-built contouring atlas and interested in the treatment of gynecological cancer were invited to participate in this 2-step trial in December 2022 during an AIRO Gynecology meeting. The fifteen colleagues who expressed a willingness to participate were contacted and provided with study materials. In the first step all participants were supplied with a DICOM format of the planning computed Tomography (CT) and the T2-weighted Magnetic Resonance Imaging (MRI) sequence of a selected clinical case of locally advanced cervical cancer (LACC). The planning-CT and MRI were performed in the supine position with raised arms, a full bladder, and an empty rectum. Participants had to identify emerging OARs (LAM: Levator ani muscle; PRM: Puborectalis muscle; IAS: Internal anal sphincter; EAS: External anal sphincter; BBT: Bladder base and trigone; BN: Bladder neck; IBM: Iliac Bone Marrow; LPBM: Lower Pelvis Bone Marrow; LSBM: Lumbosacral Bone Marrow) based on their own personal knowledge of pelvic anatomy and experience. A uniform nomenclature was adopted for each OAR to facilitate the evaluation. The suggested OARs and the contouring process were then presented at a subsequent AIRO Gynecology webinar meeting with a contouring laboratory. Finally, in the second step, each participant who had joined the study received the custom-built contouring atlas with anatomical boundaries for each emerging OAR and was requested to delineate again the OARs using the tool provided. This step took place after the webinar meeting.

All participants were given a questionnaire to complete as part of the qualitative study. This study has been evaluated by the Scientific Committee and Board of the Italian association of radiotherapy and clinical oncology (AIRO) for the critical revision and final approval of the paper.

### Measurements

Three different radiation oncologists [AA, LC, AV] examined the delineations of OARs. All three radiation oncologists have several years of experience in contouring [16] and interobserver variability studies [17], [18]. For the study, investigators measured 4 different interobserver variability parameters.

### Primary end-point

The goal of this study was to provide straightforward instructions for everyday practice in outlining emerging OARs of the female pelvis and to discuss the interobserver variability in a two-step multicenter study using as the benchmark the custom-built contouring atlas with anatomical boundaries for each emerging OAR.

### Statistical analysis

All contours for each OAR were imported into the MIM-MAESTRO program (MIM Program Inc. Cleveland OH), and various analyses were run to evaluate interobserver variability to the benchmark, as in our previous studies [17], [18]. Using the Dice similarity coefficient (DSC), the spatial overlap accuracy of the different volume delineations was evaluated and compared to the benchmark [19]. A similar statistic known as the Jaccard Similarity Coefficient (JSC) was developed by comparing the intersection of two volumes to their union [20]. For both indices, the greater the overlap between the two volumes, the lower the value must be. The Hausdorff distance (HD), defined as the maximum distance between each voxel in the reference set and the nearest point in the comparison set, was calculated to investigate the separation between contours [21]. The mean distance that each outlying point in the volume under consideration must be shifted to obtain complete conformity-overlap with the reference volume (or mean distance to agreement, or MDA) was also determined [22]. Lower values (in mm) indicate a higher degree of correlation between the compared volumes for both HD and MDA. Standard deviation (SD) and sample mean were used to describe all results. Statistical analysis was performed using Microsoft Excel 2010 and SPSS Statistic version 23 (IBM Corp.Armonk, USA).

## Results

### Contouring atlas

Experts in pelvic imaging developed the contouring atlas, which served as the quality standard for the subsequent research. It included anatomical limits and imaging resources for each emerging OAR of female pelvis. The definition of anatomical boundaries was performed as follows for all substructures and emerging OARs identified on T2-weighted MRI sequence, except for bone marrow structure delineated on planning-CT.

1. The Levator ani muscle (LAM) is the musculotendinous sheet that forms most of the pelvic floor, involved in urinary and fecal evacuation as well as maintaining continence. It appears *iso*-hypointense on T2-sequence and had the typically V-shaped form on axial plan, arising from the most cranial border of pubic bone superiorly, continuing with puborectalis muscle and anorectum medially, and the dorsal part of pubic bone, the obturator muscle fascia and the Ischiorectal fossa (IRF) laterally (Fig. 1a–d).

**Fig. 1:**
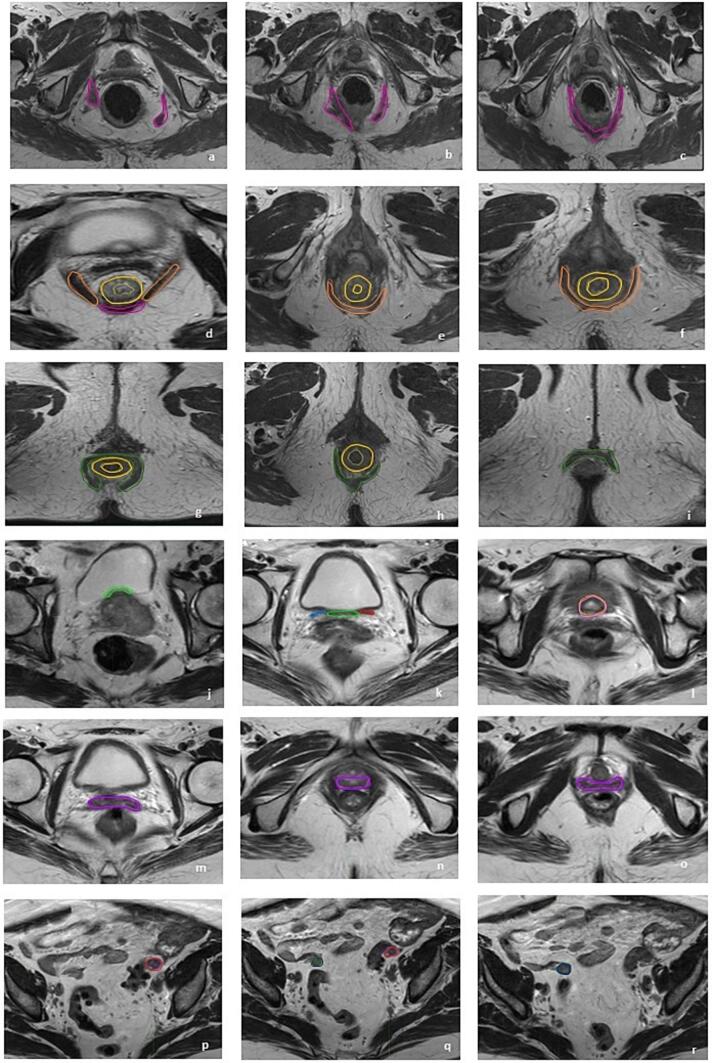
A graphical representation of the anatomical boundaries of all substructures and emerging OARs on axial planes of T2-weighted sequence. a-d: Levator ani muscle (LAM, **fuchsia**); d-f: Puborectalis muscle (PRM, **orange**); d-h: Internal anal sphincter (IAS, **yellow**); g-i: External anal sphincter (IAS, **forest green**); j-k Bladder base and trigone (**green**) between right (**sky blu**) and left ureter (**red**); l: Bladder neck (**pink**); m-o: Vagina (**violet**); p-r: right (**blu**) and left (**brown**) Ovary.
2. The Puborectalis muscle (PRM) is the ventromedial part of the LAM. It appears *iso*-hypointense on T2-sequence and on axial plan as a U-shaped muscle, which forms a sling around anorectal junction and vagina, creating the anorectal angle. It is inserted to the pubic bone passing beside the urethra, vagina, and anorectum (Fig. 1d–f). It is better visible in coronal section where PRM has greater thickness than external anal sphincter (EAS). It arises from LAM at the level of anorectal junction, with internal anal sphincter (IAS) medially and the dorsal part of pubic body and ischium-rectal fossa laterally.
3. The Internal anal sphincter (IAS) is the innermost muscle layer of anal canal and the distal continuation of the smooth muscle layer of the rectum. It appears with an intermediate signal intensity that is *iso*-hypointense on T2-sequence. It is better visualized in sagittal plane arising from anorectal junction till 1 cm above anal verge, concentrically surrounded by EAS, delimiting the anal mucosa. In the cranial portion of anal canal has a donut shape and the lumen is visible with a 2–3 mm thickness. Instead in the caudal part, where the lumen is not visible, the IAS delineation is represented by the entire section of anal canal (Fig. 1d–h).
4. The External anal sphincter (EAS) is the outermost muscle of the distal anal canal, and it extends 1 cm beyond the IAS with a thickness of 4 mm. It appears hypointense on T2 sequence, and it arises from PRM, since the deep part is fused or closely related to the PRM, till the anal orifice. It partially encircles the IAS, separated by the inter-sphincteric spaces (Fig. 1g–i).
5. The bladder base may be identified in sagittal and coronal planes as the posterior-inferior wall of the bladder, where the detrusor extends posteriorly and down into the neck. Internally, it corresponds to the trigone, which appears hypointense and is contained between the openings of the two ureters into the bladder wall on an axial view. Both the bladder base and the trigone are contoured in a single volume (Fig. 1j–k).
6. The neck of the bladder is defined as the area where the bladder wall is narrower and thicker (Fig. 1i). It has a characteristic hypo-intense funnel-shaped appearance, best recognized on sagittal planes, between the thickened wall bladder and urethra, anterior to the vagina and posterior to the symphysis pubis***.***
7. The vagina is a midline fibromuscular tubular organ with an average length of approximately 7–10 cm. On high-resolution T2-weighted sequences, the collapsed vagina appears as an hyperintense H-shaped or W-shaped configuration on axial views, as the anterior and posterior walls less rigid than lateral ones are imprinted by the surrounding pelvic structures. Three vaginal parts may be identified: upper (between cervical orifice and urethral-bladder junction), middle (between urethral-bladder junction and posterior-inferior border of the symphysis-PIBS) and lower third (between PIBS and vaginal introitus). It is bordered posteriorly by the mesorectum, rectum, and anal canal. It comes into touch with the urethra, bladder, and adipose tissue anteriorly, and the puborectalis muscle laterally (Fig. 1m–o).
8. The ovaries are usually located in the homonymous fossa, at the level of common iliac bifurcation, anteriorly and medially to ureters and iliac vessels and laterally to uterine body. In axial planes, suspensor ligament of the ovaries may be identified from the ovary to the lateral pelvic wall. The right ovary is usually medial to ileo-cecal junction, caecum, and appendix. The left ovary is adjacent to the sigmoid colon. They face the peritoneum posteriorly. (Fig. 1p–r). The aspect of the ovary’s changes in accordance with age and hormonal status of the patient. During fertile age, its identification is facilitated by the presence of follicles, resulting hyperintense in T2 weighted sequences. In menopausal, they appear hypointense in T1 and T2 weighted sequences.
9. The Pelvic bones were delineated on the bone window of the planning-CT scan as a surrogate of whole Pelvic Bone Marrow (PBM) and then divided into 3 subsites: the iliac BM (IBM), extending from the iliac crests to the upper border of femoral head; the lower pelvis BM (LPBM), accounting for bilateral pubic rami, ischium, acetabulum and proximal femur, from the upper limit of the femoral heads to the lower limit of the ischial tuberosities; lumbosacral BM (LSBM), extending from the superior border of L5 (5th Lumbar Vertebra) somatic body to the entire sacrum.

A graphical representation on axial planes is shown in Fig. 1.

### Qualitative analysis

Fifteen radiation oncologists from various institutions participated in the study and provided the two-times outlined volumes. Table 1 provides a full summary of the qualitative questionnaire's findings. The engagement of Institutions included almost all of Italy's macroregions (North, Center, and South). With the exception of one resident, the majority of the population were radiation oncology professionals. 87 % of radiation oncologists with a focus on gynecological cancer are highly skilled and have a professional seniority of over 10 years. The majority of centers treated more than 10 cases annually, and 27 % of them reported treating more than 20 cases. The interdisciplinary tumour board had a major effect on the clinical choices.

**Table 1:** Qualitative analysis results (*multiple-choice question).

**N⁰ of questionnaires:15 (%)**	**RT Center (Italy)**			**Professional seniority**			**Resident**	
	**North**	**Center**	**South**	**< 5 yr**	**5**–**10 yr**	**>10 yr**	**Yes**	**No**
	5 (33)	6 (40)	4 (27)	5 (33)	2 (13)	8 (53)	1 (7)	14 (93)
	**Expertise in Gynecological Malignancy**		Tumor board support (*)			**N⁰ treatment/year**		
	**Yes**	**No**	**Always**	**Selected cases**	**Rarely**	**< 10**	**10**–**20**	**>20**
	13 (87)	2 (13)	3 (20)	7 (47)	5 (33)	3 (20)	8 (53)	4 (27)

### Interobserver variability and volume parameters

**Fig. 2:**
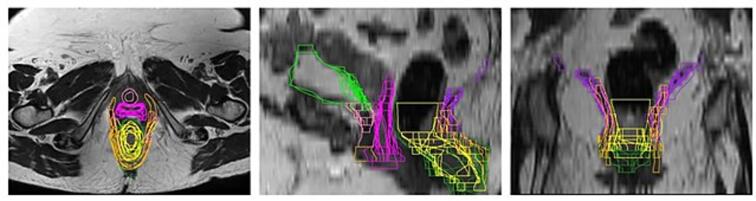
A graphical representation of interobserver variability among centres of all substructures and emerging OARs on axial (a), sagittal (b), and coronal (c) planes of T2-weighted sequence: Levator ani muscle (LAM, fuchsia); Puborectalis muscle (PRM, orange); Internal anal sphincter (IAS, yellow); External anal sphincter (IAS, forest green); Bladder base and trigone (green); Bladder neck (pink); Vagina (violet).

Descriptive volumes analysis and the evaluated indices are detailed in Table 2. The participants had a high degree of agreement for pelvic bones sub-structures (IBM, LPBM, LSBM) delineation on planning CT. Regarding the anal-rectal sphincter complex, the higher agreement was reported for EAS, with a mean DCS of 0.605 ± 0.05 and MDA of 1.61 ± 0.25 mm, whereas the agreement was low with a high degree of interobserver variability for the other sub-structures of the anal-rectal sphincter complex (LAM, PRM, and IAS). Indeed, maximum HD values for LAM and PRM were larger than 30 mm (Table 2). The agreement resulted moderate for bladder base and trigone (mean DCS: 0.515 ± 0.08 and MDA: 1.54 ± 0.48 mm) and low for bladder neck (mean DCS = 0.515 ± 0.08 and MDA = 2.21 ± 1.10 mm). Finally, a moderate degree agreement was showed for ovaries (right ovary: mean DSC = 0.61 ± 0.16 and MDA = 2.78 ± 1.37 mm; left ovary: mean DSC = 0.72 ± 0.05 and MDA = 1.3 ± 0.29 mm) and vagina (mean DSC = 0.575 ± 0.13 and MDA = 3.23 ± 1.37 mm). Fig. 2 displays a visual representation of interobserver variation across centers.

**Table 2:** Descriptive volumes analysis and evaluated indices. Legend: LAM: Levator ani muscle; PRM: Puborectalis muscle; IAS: Internal anal sphincter; EAS: External anal sphincter; BBT: Bladder base and trigone; BN: Bladder neck; R: right; L: left; IBM: Iliac Bone Marrow; LPBM: Lower Pelvis Bone Marrow; LSBM: Lumbosacral Bone Marrow; DCS: Dice similarity coefficient; JCS: Jaccard Similarity Coefficient; HD: Hausdorff distance; MDA: mean distance to agreement.

**T2 Weighted MRI**										
	**Volume (cm^3^)**		**DSC**		**JSC**		**Max HD (mm)**		**MDA (mm)**	
	**Mean ± SD**	**Range**	**Mean ± SD**	**Range**	**Mean ± SD**	**Range**	**Mean ± SD**	**Range**	**Mean ± SD**	**Range**
**LAM**	16.6 **±** 6.54	4.71––29.03	0.345 **±** 0.07	0.24–0.49	0.21 **±** 0.05	0.13–0.33	32.65 **±** 1.47	30.25–35.42	6.13 **±** 1.34	4.23–9.15
**PRM**	12.39 **±** 5.65	6.12–29.0	0.41 **±** 0.10	0.21–0.50	0.26 **±** 0.08	0.11–0.33	33.61 **±** 3.40	25.70–37.85	4.26 **±** 2.07	3.02–9.54
**IAS**	17.93 **±** 7.98	10.87–37.89	0.4 **±** 0.07	0.27–0.5	0.25 **±** 0.047	0.2–0.34	22.02 **±** 8.21	0.96–32.59	4.31 **±** 1.84	1.63–7.32
**EAS**	19.09 **±** 5.56	15.01–32.77	0.605 **±** 0.05	0.5–0.66	0.44 **±** 0.05	0.33–0.49	14.5 **±** 4.08	11.75–24.31	1.61 **±** 0.25	1.35–2.23
**BBT**	3.92 **±** 4.54	1.71–8.46	0.515 **±** 0.08	0.4–0.66	0.345 **±** 0.08	0.24–0.49	7.37 **±** 2.13	6.01–12.52	1.54 **±** 0.48	1.19–2.81
**BN**	3.61 **±** 1.80	1.91–8.13	0.49 **±** 0.18	0.24–0.84	0.32 **±** 0.17	0.13–0.72	8.05 **±** 3.09	4.02–14.29	2.21 **±** 1.10	0.64–4.06
**Vagina**	22.4 **±** 9.10	7.39–44.5	0.575 **±** 0.13	0.38–0.81	0.395 **±** 0.13	0.23–0.67	17.895 **±** 5.65	7.47–28.71	3.23 **±** 1.37	1.24–5.98
**Ovary R**	3.095 **±** 1.59	1.58–6.87	0.61 **±** 0.16	0.4–0.83	0.43 **±** 0.17	0.25–0.71	15.45 **±** 5.09	4.97–18.73	2.78 **±** 1.37	0.81–4.38
**Ovary L**	4.08 **±** 1.04	2.29–6.85	0.72 **±** 0.05	0.62–0.79	0.56 **±** 0.06	0.45–0.65	6.61 **±** 1.78	4.02–10.47	1.3 **±** 0.29	0.89–1.84
**Planning CT**										
**IBM**	371.02 **±** 19.76	355.64–412.83	0.9 **±** 0.02	0.86–0.94	0.82 **±** 0.03	0.76–0.88	7.38 **±** 2.04	4.14–11.06	0.77 **±** 0.15	0.48–1.00
**LPBM**	510.23 **±** 22.15	465.98–557.27	0.91 **±** 0.01	0.88–0.94	0.84 **±** 0.02	0.79–0.88	14.59 **±** 1.81	0.85–16.81	0.92 **±** 0.14	0.68–1.24
**LSBM**	350.32 **±** 37.54	257.72–391.77	0.79 **±** 0.03	0.76–0.86	0.65 **±** 0.04	0.61–0.76	27.28 **±** 3.77	20.39–33.96	2.91 **±** 0.47	1.75–3.47

## Discussion

The selection and definition of OARs are key steps in delivering precise and tailored RT and reducing toxicity. Nowadays, guidelines and contouring atlases for OARs delineation are commonly available and adopted. A large review of the anatomical definitions of many and emerging OARs, imaging modalities required for their definition, and dose/volume constraints was recently completed [23]. On the other hand, common indication for sub-structures delineation and their boundaries definition are still lacking, leading to inhomogeneous contours. Several strategies can be implemented to reduce contouring uncertainties, as quality assurance processes, the use of shared delineation guidelines, and periodic training for radiation oncologists.

In this context, we proposed, together with the Italian AIRO group for gynecological malignancies, practical instructions for sub-structures and emerging OARs delineation of female pelvis, to facilitate their contouring in daily practice. In order to emphasize this pertinent and important issue, a study of the interobserver variability was also done. The DSC and the JSC were used as the most common measures for the geometric quantification of contour similarities. Despite their popularity, these conventional volumetric overlap indices may not predict the clinical adequacy of contours and has reported to provide limited correlation with clinical or dosimetric quality in brachytherapy planning for cervical cancer [24]. To overcome the limitations of volume-based metrics, spatial distance-based metrics (HD, as the maximum surface distance, and MDA as mean distance to agreement) which are more sensitive to boundary errors, were calculated, as suggested by some authors [20], [25].

To the best of our knowledge there are no other studies that define anatomical boundaries of the anal-rectal sphincter complex and evaluate the contouring variability of each substructure involved in normal fecal continence [26], [27]. The higher agreement was reported for EAS, while a low uniformity has been reported for LAM, PRM, and IAS. This could be related to the small volumes of the outlined sub-structures and the paucity of knowledge about their anatomical boundaries by the radiation oncologist, given that guideline for the definition, identification and contouring of these sub-structures have not yet been provided and their delineation is not yet a routinely practice. However, the importance of recognize and correctly delineate the anal-rectal sphincter complex is of utmost importance, because overdosage could result in alterations of morphology and function of the female pelvic floor muscles, leading to weakness of the external anal sphincter, stiffness of the rectal wall, and a consequent increase in rectal sensitivity [11]. Several reports on radiation therapy for prostate cancer have described the relationship between dose-volume parameters for the rectum and anal canal and late anal-rectal toxicity, and evidence has been provided that various incontinence-related complaints may originate from specific anorectal subareas [28], [29]. Smeenk et al. delineated the LAM, the PRM, the IAS, and the EAS in 48 patients treated for localized prostate cancer and reported a dose–effect relationships for individual pelvic floor muscles and anorectal complaints (fecal urgency) after prostate radiotherapy [12].

In regard to the delineation of urinary tract substructures (bladder base, trigone and bladder neck), in our study, the interobserver agreement resulted modest for bladder base and trigone and low for bladder neck. Indeed, some studies of radio(chemo)therapy for prostate cancer [30], [31] and for cervical cancer [13], [14] have shown that some urinary tract substructures are responsible for urinary morbidity and that the dose to the bladder base is associated with urinary tract obstruction, frequency, urgency, dysuria, and incontinence. Unfortunately, CT-simulation imaging was used for treatment plans in these studies, and a clear definition of the sub-structures was not reported. A review of literature regarding anatomy, physiology, and imaging of the lower urinary tract was recently conducted and the sub-structures potentially involved in radiation-induced injuries (trigone, bladder neck and urethra) were identified on MRI. Then, a contouring consensus between radiologists, radiation oncologists and uro-gynecologist was proposed and intra-observer consistency was tested on 210 MRIs for Image-Guided Adaptive Brachytherapy (IGABT) in 105 LACC patients treated with radiochemotherapy [32], [33]. The median volumes of the bladder, trigone and bladder neck measured on MRI were assessed. The greatest variation occurred in bladder volume and bladder neck, which is related to the influence of bladder filling, while the height and width of the trigone showed the smallest variation between the two IGABT fractions [32], [33]. In our study, the mean volume of the bladder base and trigone was almost comparable to that reported by Spampinato S. et al (3.92 ± 4.54 cm^3^ compared to 4.0 cm3), while the bladder neck was significantly smaller (3.61 ± 1.80 cm3 compared to 9.8 cm^3^) [32]. This difference could be explained by the lack of a distinguishable contrast on MRI of the bladder neck and the difficulty in clearly defining its superior border in relation to anatomic references [33].

A moderate degree interobserver agreement was showed for the vagina, confirming previous studies showing a rather large variability in upper and lower border on MRI with a mean DSC of 0.43 [34], [35]. In our analysis a higher agreement was reported for vagina since a mean DSC of 0.575 ± 0.13 and an MDA of 3.23 ± 1.37 mm, probably related to the improved knowledge in vagina delineation acquired in the last years. In fact, the introduction of IGABT leads to detailed delineation of target volumes and OARs, dose reporting according to previously established goals and constraints, and the elimination of Point A prescribing [5], [6], [7]. Vaginal toxicity is an important and feared drawback of pelvic brachytherapy, especially in patients in whom both cure rate and predicted survival are high. Therefore, late vaginal toxicity should be avoided in modern radiotherapy, which pays attention to the quality of life and patient-reported outcomes [15]. In the era of adaptive brachytherapy such precise contouring together with NTCP models [36] to estimate vaginal toxicity and dose constraints to avoid severe vaginal late effects [37], [38], [39], could overcome these challenges. Moreover, in the context of building models for predicting toxicity endpoints, a radiomic approach based on, for example, Dose Volume Histogram (DVH) metrics, and treatment-related clinical variables has also been considered [40]. Planning-based DVH constraints and dosimetric radiomics analyses could be promising tools for personalizing radiotherapy planning.

As per ovaries delineation uniformity, in our analysis, it resulted quite high since a mean DSC of 0.61 ± 0.16 (right) and 0.72 ± 0.05 (left) with an MDA of 2.78 ± 1.37 mm (right) and 1.3 ± 0.29 mm (left). However, the ovaries are very radiosensitive organs and are generally not considered OAR in gynecologic planning. On the contrary, ovarian-sparing planning techniques have been evaluated for soft tissue sarcomas of the buttock and thigh [41]. For this purpose, due to significant internal movement and poor location reproducibility [42], a diagnostic MRI of the pelvis should be performed to determine the ovaries’ location [41].

Finally, chemoradiation for LACC patients can induce bone marrow (BM) suppression [43], [44]. The multi-institutional phase II INTERTECC-2 trial used IMRT to spare BM and a subset of the trial used an optional PET/CT to delineate functional BM for contouring to help reduce myelosuppression [3]. Knowledge on the spatial location of BM is essential for the development of BM sparing RT techniques and several other methods for BM delineation have been evaluated in clinical trials [45]. Several studies analyzed the correlation between three subsites of the pelvic bone and hematologic toxicity [3], [46], [47] and dose constraints have been proposed. Based on these experiences, the variability of delineation on CT-simulation of Iliac, Lower Pelvis, and Lumbosacral Bone Marrow, was analyzed in our study. The higher consistency of homogeneity was reported for pelvic bones compared to all other OARs or sub structures. This good result is likely related to the routine use of CT in delineating bone structures and the widespread practice of delineating pelvic bone as OAR in radio chemotherapy for cervical cancer, which underscores the need for a learning curve in delineating target volumes and OAR in radiotherapy planning.

A drawback of the study is that the degree of agreement was tested on only one clinical case and needs to be validated in clinical trials and daily practice. The use of numerous cases could better demonstrate the reliability and reproducibility of the proposed delineation atlas because of the anatomic differences between patients. In addition, no expert consensus on the proposed atlas has been performed yet, which could be the next step of the study.

## Conclusion

This study provides straightforward instructions for daily routine in delineating emerging OARs of the female pelvis and report a moderate to low level of agreement in the delineation of the female pelvis emerging OARs, with a high degree of variability among observers. Defining such structures could become part of our daily OAR contouring process as increasingly precise control of acute and late pelvic toxicity is required. The development of gynecologic radiotherapy Normal Tissue Complication Probability models, artificial intelligence atlases, guidelines, and contouring tools should be encouraged to improve the routine contouring of these OARs and increase the quality and consistency of radiotherapy planning.

## Patient consent

Not applicable because non-identifiable images.

## Funding

This research received no external funding.

## CRediT authorship contribution statement

**A. Augurio:** Conceptualization, Methodology, Collection, Analysis and interpretation of data, Validation, Writing—original draft preparation. **G. Macchia:** Conceptualization, Methodology, Writing—review and editing. **L. Caravatta:** Collection, Analysis and interpretation of data, Validation, Writing—original draft preparation. **M. Lucarelli:** Collection, Analysis and interpretation of data. **F. Di Gugliemo:** Collection, Analysis and interpretation of data. **A. Vinciguerra:** Collection, Analysis and interpretation of data. **B. Seccia:** Conceptualization, Methodology. **V. De Sanctis:** Collection, Analysis and interpretation of data. **R. Autorino:** Collection, Analysis and interpretation of data. **C. Delle Curti:** Collection, Analysis and interpretation of data. **S. Meregalli:** Collection, Analysis and interpretation of data. **E. Perrucci:** Collection, Analysis and interpretation of data. **D. Raspanti:** Collection, Analysis and interpretation of data. **A. Cerrotta:** Conceptualization, Methodology, Validation, Writing—review and editing.

## Declaration of Competing Interest

The authors declare that they have no known competing financial interests or personal relationships that could have appeared to influence the work reported in this paper.
